# Depression and the risk of fibromyalgia syndrome: a two-sample Mendelian randomization study

**DOI:** 10.3389/fpsyt.2024.1282172

**Published:** 2024-11-12

**Authors:** Xiaoshan Ma, Jing Sun, Ren Geng, Yao Zhao, Wanzhen Xu, Yining Jiang, Liyan Zhao, Yunqian Li

**Affiliations:** ^1^ Department of Neurosurgery, First Hospital of Jilin University, Changchun, China; ^2^ Department of Anesthesiology, First Hospital of Jilin University, Changchun, China; ^3^ Department of Neurosurgery, Qilu Hospital of Shandong University Dezhou Hospital, Dezhou, China; ^4^ Department of Blood Transfusion, Second Hospital of Jilin University, Changchun, China

**Keywords:** depression, fibromyalgia, single nucleotide polymorphism, risk, Mendelian randomization analysis

## Abstract

**Background:**

Fibromyalgia (FM) is a common illness with a wide range of symptoms, mainly manifested by unexplained chronic systemic musculoskeletal pain, sleep disorders and fatigue, sometimes accompanied by cognitive impairment, psychiatric symptoms and autonomic dysfunction. Previous studies have indicated a correlation between depression and the risk of FM; however, it remains uncertain whether this association reflects a causal relationship.

**Methods:**

We evaluated the etiological association between the genetically predicted depression and the risk of developing FM by conducting a two-sample Mendelian Randomization (MR) study. The data on single nucleotide polymorphisms (SNPs) related to depression were obtained from the UK Biobank (UKB) and the Psychiatric Genomics Consortium (PGC) of White British European ancestry, and the data for FM were from the 5th release of the FinnGen study. We adopted the Inverse Variance Weighted (IVW) approach as the principal standard. In order to detect the existence of horizontal pleiotropy and heterogeneity, we adopted the MR-Egger approach as the sensitivity analysis.

**Results:**

In our MR analysis, 42 depression-related variants were identified as valid instrumental variables (IVs). The IVW approach’s results manifest that there is no etiologic causality between genetically predicted depression and the risk of FM (odds ratio [OR]: 1.673, 95% confidence interval [CI]: 0.852—3.287, *P* = 0.135). The study did not find any significant heterogeneities or horizontal pleiotropies (*P* > 0.05).

**Conclusions:**

Our results suggest that there is no significant genetic evidence linking depression to an increased risk of FM. However, further research is necessary to investigate the potential relationship and underlying mechanisms between depression and the risk of FM.

## 1 Introduction

Fibromyalgia (FM) is a distressing syndrome that is characterized by chronic widespread musculoskeletal pain, hyperalgesia and psychological distress (1). The prevalence and gender distribution of FM are closely related to the diagnostic criteria and the doctor’s confirmation bias, as well as the number and severity of symptoms. Different diagnostic criteria will lead to different results (2). The doctor’s confirmation bias is more likely to occur in women, and the number and severity of symptoms are significantly higher in women than in men (2, 3). The prevalence of FM in the general population is about 0.2-6.6%, and the incidence of FM in existing studies is higher in women than in men to varying degrees (2–4). Patients with FM typically experience a wide range of symptoms, including local pain syndromes, gastrointestinal disorders, stiffness, cognitive impairment, psychological distress, autonomic nerve disorders, or gait instability (5). This heterogeneity in symptoms experienced by individuals can often result in decreased levels of daily functioning, productivity at work, and overall health-related quality of life (6).

Fibromyalgia is frequently found to coexist with other conditions or diseases, such as temporomandibular disorders, irritable bowel syndrome (IBS), depression, headaches, and mood disorders (7). Depression is a widespread mood disorder characterized by euphoria deficiency and persistent sadness, accompanied by noticeable psychological and nutritional changes such as somnolence, loss of motivation, difficulty maintaining attention, anorexia and guilt, and even suicidal behavior, affecting approximately 1.7 times more women than men (8). People with depression may exhibit medically unexplained somatic symptoms, including back pain, abdominal pain, and headaches, with the prevalence of pain estimated to be around 65%, which is significantly higher than the 29% observed in the non-depressed population (9). An early study from Finland suggests that depressive symptoms serve as a predictor for the future occurrence of musculoskeletal symptoms (10). A recent study in Brazil found that a comprehensive healthcare model combining systematic health education with physical activity significantly improves the health status of patients with FM (11). Recent longitudinal clinical and community-based studies have yielded suggestive evidence of an interrelationship between chronic pain and depression (12–15). A bidirectional association has been observed between FM and depression: depression increases the risk of diagnosing FM later in life, and FM increases the risk of developing depression (16). Research from both Europe and North America has drawn the same conclusion: approximately 30% of patients are already experiencing major depressive disorder (MDD) at the time of their initial FM diagnosis, and the severity of FM is positively correlated with the severity of their depression (17, 18). A case-control study found that MDD patients had elevated serum levels of several proteins involved in controlling muscle function, and that these proteins were highly positively correlated with the frequency of some of the major symptoms in the FM syndrome diagnostic scale (19). However, the design of previous findings was observational, which does not allow us to conclude causality. Addressing these causal questions could accelerate the discovery of underlying disease mechanisms and open up new avenues of prevention and treatment.

Mendelian randomization (MR) is a method that can determine the causal relationship of certain environmental exposures by utilizing the random classification of genes from parents to offspring during gamete formation and pregnancy (20). The MR method employs genetic variations, which are determined at conception, as instrumental variables (IVs) to explore the influence of exposure factors on the risk of specific diseases. Because IVs mentioned above are typically irrelevant to any confounders, the MR method can more reliably explore the potential causality between exposure factors and outcomes of some diseases. Meanwhile, since the segregation of alleles is carried out in a random way, the MR method can also minimize the bias generated by confounders (21).

Previous MR Studies have not explored the causal relationship between depression and FM. This research adopted a two-sample MR approach to investigate the association between depression and the risk of developing FM by analyzing genome-wide association study (GWAS) databases.

## 2 Materials and methods

### 2.1 Data resources

For depression, summary GWAS results were obtained from the UK Biobank (UKB) and the Psychiatric Genomics Consortium (PGC) of White British European ancestry, which included 170756 cases and 329443 controls (22). The establishment of a diagnosis of depression and the evaluation of its severity are based on the International Classification of Diseases (ICD-10) and the American Psychiatric Association’s Diagnostic and Statistical Manual (DSM-IV) (23). The dataset of extensive depression phenotype may comprise a variety of other psychiatric disorders, while a clinically diagnosed MDD phenotype can provide a more credible definition of depression (22). As a supportive instrument for investigating the risk of serious diseases, the UKB successfully recruited men and women between the ages of 40 and 69 from 2006 to 2010 and tracked their health status for an extended period, with a total of 500,000 people (24). PGC is the largest and most innovative consortium in the field of psychiatry, and it has unified most of the content in the field. Researchers can use it to conduct large-scale analysis of GWAS data on psychiatric disorders (25). The primary outcome for this study was FM. Summary statistic data for FM were acquired from the 5th release of the FinnGen study, which included 737 cases and 167,641 controls of European individuals. The FinnGen study is a research project composed of several research institutions, hospitals and biological companies, which combines the health registration data collected from every resident over the age of 18 in Finland since 1969 with the new samples collected from the Finnish Biobank, and has so far generalized samples from approximately 400,000 people and analyzed more than 200,000 people (26). **Table 1** shows the details about the datasets.

**Table 1:** Characteristics of data sources and strength of IVs used in the Mendelian Randomization study.

Exposures/Outcomes	Consortium	Ethnicity	Sample Sizes	F-Statistic (Total)
**Depression**	UK Biobank and PGC	European	500,199	NA
**FM**	FinnGen Biobank	European	168,378	16.38402146

FM, fibromyalgia; NA, Not Available; PGC, the Psychiatric Genomics Consortium.

### 2.2 Selection and characterization of associated IVs

Independent genetic variants with depression were chosen to be genetic IVs. The single nucleotide polymorphisms (SNPs) selected in this study were strongly correlated with exposure factors, reaching the genome-wide significance criterion (*P*< 5 × 10^–8^). Furthermore, we identified the corresponding linkage disequilibrium for SNPs and used criteria defined by r2<0.001 and clustering window =10,000kb to eliminate deviations triggered by linkage disequilibrium. Then, we have screened and eliminated some SNPs related to potential confounders (http://www.phenoscanner.medschl.cam.ac.uk/). These confounding factors can affect the results of MR analysis. In this study, we ascertained chest pain, rheumatoid arthritis, systemic lupus erythematosus and Sjogren’s syndrome as confounders (27). We conducted the harmonization between SNPs of exposure and outcome to ensure that the orientation of the alleles can be rectified (28). R-square and the F-statistic values were calculated to mitigate the bias and assess the strength of IVs (29, 30). It is generally considered to exclude the bias of weak IVs when the F statistic >10.

### 2.3 Statistical analysis

In this study, we adopted the Inverse Variance Weighted (IVW) approach as the principal standard (31). We adopted Cochran’s Q test to examine the heterogeneity between individual genetic variation estimates. We adopted the fixed-effects model as the ultimate consequences of MR Study, when the *P*-value of Cochran’s Q test was greater than or equal to 0.05; otherwise, the random-effects model of IVW was adopted (32). In order to supplement the analysis of IVW and increase the reliability of the analysis results, we also used maximum likelihood, weighted median method (WM), and penalized weighted median method (PWM) (33, 34). Furthermore, we adopted a newly proposed MR analysis termed robust adjusted profile score (RAPS). It can regulate horizontal pleiotropy and decrease the deviation triggered by horizontal pleiotropy (35). Finally, we certify the consequences of the IVW method through MR Pleiotropy residual sum and outlier (MR-PRESSO) (36).

### 2.4 Sensitivity analysis

To confirm the possibility of pleiotropy, we adopted the MR-Egger model. When the *P*-value for intercept of the MR Egger model is not less than 0.5, it manifests that horizontal pleiotropy does not exist (37). We use R software to create a plot of the leave-one-out sensibility test by eliminating every independent SNP every time. It can be used to evaluate whether these consequences are stable. We further developed funnel plots and forest plots as supplementary means for detecting pleiotropy. Each *P*-value is two-sided, and when the *P*-value is less than 0.05, it indicates the presence of suggestive significance. Each analysis and detection in this study was completed on the basis of applying the “Two-Sample-MR”, “MR-PRESSO” and “MR. RAPS” packages in R software (version number 4.2.2).

## 3 Results

### 3.1 IVs selection

**Table 1** presents the specific situation of the IVs representing depression in this study. We adopted 42 SNPs as IVs for depression on FM (**Supplementary Table 1**). Our analysis results indicate that there is no existence of weak instrumental variable bias (F-statistic > 10) (**Table 1**).

### 3.2 Association of depression with FM risks

**Table 2** summarizes the results of our MR analysis on the causal association between depression and FM risks. Using the fixed-model IVW method, we found no significant evidence that depression is associated with an increased risk of FM (OR: 1.673, 95% CI: 0.895-3.127, *P*= 0.107). Our results were consistent with various MR methods, including maximum likelihood, MR-RAPS, weighted median, and penalized weighted median methods. Furthermore, the consistency of the MR-PRESSO causal estimation values demonstrates that our research findings are both highly reliable and stable (**Table 3**).

**Table 2:** Mendelian randomization for depression on the risk of Fibromyalgia.

	Weighted Median	IVW (random effects)	Maximum likelihood	Penalised weighted median	IVW (fixed effects)	MR-RAPS
**OR Estimate (95% CI)**	1.098 (0.415-2.906)	1.673 (0.852-3.287)	1.707 (0.905-3.221)	1.025 (0.394-2.665)	1.673 (0.895-3.127)	1.767 (0.858-3.637)
***P*-Value**	0.851	0.135	0.099	0.959	0.107	0.122
**Beta**	0.093	0.515	0.535	0.025	0.515	0.569

OR, odds ratio; CI, confidence interval; IVW, inverse variance weighted; MR-RAPS, Mendelian Randomization robust adjusted profile score.

**Table 3:** MR-PRESSO for causal effect between Depression and Fibromyalgia.

Outcome		Raw Estimates			Outlier Corrected Estimates				Distortion Test
	nSNP	Beta	OR (95%CI)	*P*-Value	nSNP	Beta	OR (95%CI)	*P-*Value	*P*-Value
**FM**	42	0.604	1.830 (0.945-3.543)	0.081	NA	NA	NA	NA	NA

FM, fibromyalgia; nSNP, the numbers of single nucleotide polymorphism; OR, odds ratio; CI, confidence interval; NA, Not Available.

### 3.3 Sensitivity analyses for MR study

The intercept value of the MR-Egger method demonstrated that there is no horizontal pleiotropy in the analysis consequences (*P* for intercept = 0.446) (**Table 4**). The results of heterogeneity testing analysis indicate that there is no potential heterogeneity in this study (**Table 4**). The scatter plot, forest plot and funnel plot for the MR study were exhibited in **Supplementary Figures 1**-**3**. The leave-one-out sensibility test indicated that the causality of depression on the risk of developing FM was not affected by a single SNP, no matter which SNP was eliminated, it could not have an essential impact on the results, underscoring the reliability of our study (**Supplementary Figure 4**).

**Table 4:** Pleiotropy and heterogeneity test for Depression on Fibromyalgia.

Pleiotropy test					Heterogeneity test					
	MR-Egger			MR-PRESSO	MR-Egger			Inverse variance weighted		
	Intercept	SE	*P*	*P*	Q	Q_df	Q_pval	Q	Q_df	Q_pval
**FM**	0.052	0.067	0.446	0.226	44.78	38	0.209	45.48	39	0.220

FM, fibromyalgia; MR-PRESSO, Mendelian Randomization pleiotropy residual sum and outlier.

## 4 Discussion

Previous literature have provided conclusion of robust correlations between depression and functional somatic disorders (FSD), including chronic fatigue syndrome (CFS), migraine, irritable bowel syndrome (IBS), and FM (38–41). There are mounting data supporting that FM is commonly accompanied by psychiatric and psychological conditions such as anxiety, decreased sleep quality, depression, and cognitive disturbance (42, 43). Approximately 30% of people with FM suffer from MDD at the time of diagnosis (18). From the perspective of psychosocial factors, the higher degree of depression have been found to be associated with greater FM severity (17). A 10-year follow-up survey found that depressive symptoms can predict the future development of various pain diseases, including low back pain, neck-shoulder pain and other musculoskeletal disorders (10). A Nationwide Longitudinal Study supported a bidirectional temporal association between depression and FM, suggesting that each disorder occurring first would have an increased likelihood of the other disorder subsequently (16). A meta-analysis of medically unexplained physical symptoms, anxiety, and depression found that FM is associated with depression but not exclusively dependent on it (44). These observational studies have some limitations, which may be due to potential confounding factors and reverse causal associations. Individuals without FM may have high-risk factors for depression exposure but have not yet been diagnosed with depression. Therefore, it is not explained from the perspective of genes whether depression itself promotes the high-risk of FM in the future or the high-risk factors of depression increase the possibility of developing to FM in the future. In this research, depression IVs pertinent to features that may affect the FM risk independent of depression were omitted to minimize the impact of confounders.

Current evidence suggests that complex underlying mechanisms are involved in FM. Several possible mechanisms have been put forward to interpret the association between depression and FM. Firstly, neuroimaging researches have demonstrated that there is a prominent overlap in brain regions with abnormal functions in both depression and FM. These regions include the amygdala, dorsolateral prefrontal complex, hippocampus, thalamus and anterior cingulate cortex (45). Secondly, the biochemical theoretical mechanisms of depression have suggested that the unbalance of monoamine transmitters (such as serotonin) neurochemical secretion and reuptake regulation forms the basis of depression, and that these neurotransmitters may have a crucial role in regulating the transmission of pain signaling molecules, leading to changes in pain perception (9). During a depressive episode, the dysregulation of serotonin and norepinephrine systems can increase the pain response by reducing the inhibitory function that modulates pain, whether top-down or bottom-up (45). In addition, when individuals suffer from anxiety neurosis and depressive disorder, the sympathetic autonomic nervous system becomes more responsive to the discomfort caused by these diseases themselves, which can amplify the pain of FM (46). This amplification may be due to vasoconstriction, as prolonged peripheral sympathetic vasoconstriction can lead to muscle ischemia, which sensitizes nociceptors and results in muscle pain (47). The diagnosis of FM is difficult to establish, and some patients have symptoms that involve almost every structure in the body (19). Patients with FM often need to see multiple doctors and it can take years to be diagnosed (1). The diagnostic criteria for FM have been continuously improved, and five different sets of diagnostic guidelines have been published since 1990 (48–51). The demand for a physical examination of tender points in specific areas (these areas also tend to be more sensitive in most normal individuals than others) made the 1990 ACR classification standard not applicable in practical situations (48, 49). The ACR criteria, published in 2010 and 2011, eliminated physical examination of tender points as a necessary diagnostic condition (49, 52). In addition to a more detailed definition of the original symptom (chronic widespread pain), the newly published criteria also extended the supportive diagnosis to other clinical manifestations, such as cognitive disturbances, psychiatric symptoms, morning stiffness, and autonomic dysfunction (50, 51). Therefore, a detailed and comprehensive history collection is essential for the diagnosis of FM. Doctors should be alert to the possibility of FM when patients have unexplained symptoms of diffuse pain, fatigue and decreased sleep quality (as referenced by the FibroFatigue scale) that persist for months (53). Previous epidemiological and clinical researches have concluded that depressive disorder may play a significant role in the occurrence and development of FM. However, our two-sample MR Analysis revealed that depression was not associated with an increased risk of FM syndrome.

Our two-sample MR study has yielded genetic evidence indicating that there is no significant association between depression and FM risks. Our research has a lot of advantages. First of all, IVs for depression and FM were obtained from dissimilar consortiums, which has enhanced the statistical level of our study to detect subtle influences in complicated models (54). Secondly, utilizing dissimilar samples has increased the total specimen magnitude, thereby improving the exactitude of causality estimates. Thirdly, rigorous criterion for the selection of IVs were developed, and only variants of depression that were prominently relevant to depression measurements and were eligible for the three primary hypotheses of MR study were used for IVs (55). Finally, heritable variations have evolved in dissimilar chromosomes, and the latent mutual effects between various genes are likely to have small influence on the estimation (56). Our research offers insights into the genetic factors associated with the link between depression and FM, allowing healthcare professionals to assess patients’ health status from a more comprehensive perspective. This approach ensures that while treating one condition, they also consider the risk or severity of the other condition. Although our research has many merits, some limitations of MR study should also be recognized. Firstly, we cannot absolutely eliminate underlying pleiotropy. It may cause deviation in the assessment of causality. However, we performed sensitivity analyses using multiple methods to obtain consistent outcomes, which let the conclusions of our analysis reassuring. Secondly, only SNPs associated with definite confounding factors were eliminated, and additional indefinite confounding factors may have impact on the correlation between depression and FM, necessitating further investigation. Thirdly, the data used in the analysis principally came from individuals of European ancestry, and disease patterns may differ across ancestries, making the generalizability of the findings to other populations uncertain. Finally, the diagnosis of depression mainly relies on the DSM and the ICD (23). And depending on the number, type, and severity of actual symptoms, depression can be classified as mild depression and MDD (8). Because the IVs used in this research were extracted from the summary data in the GWAS, we were unable to detect an association between the mild depression subgroup and the risk of FM. However, we anticipate conducting more extensive GWAS studies in the future to address these limitations.

In this two-sample MR analysis, we observed evidence that genetically predicted depression was not causally associated with an increased risk of FM in European population. Considering the relative scarcity of research on this subject, our findings provide significant insights into managing and preventing the disease and controlling and assessing progression. Our study used existing GWAS databases to identify an association between depression and FM, which contributes to epidemiology and clinical practice to some extent.

## 5 Conclusion

In summary, our MR analyses have provided genetic evidence that genetically determined depression is not significantly associated with the risk of FM. This was the first two-sample MR investigation into the causal effect of depression on FM. Further large-scale randomized controlled trials are required to substantiate our research conclusion.

## Data Availability

The original contributions presented in the study are included in the article/**Supplementary Material**. Further inquiries can be directed to the corresponding authors.

## References

[1] Bourke SL Schlag AK O'Sullivan SE Nutt DJ Finn DP. Cannabinoids and the endocannabinoid system in fibromyalgia: A review of preclinical and clinical research. Pharmacol Ther. (2022) 240:108216. doi: 10.1016/j.pharmthera.2022.108216 35609718

[2] Wolfe F Walitt B Perrot S Rasker JJ Häuser W. Fibromyalgia diagnosis and biased assessment: Sex, prevalence and bias. PLoS One. (2018) 13:e0203755. doi: 10.1371/journal.pone.0203755 30212526 PMC6136749

[3] Srinivasan S Maloney E Wright B Kennedy M Kallail KJ Rasker JJ. The problematic nature of fibromyalgia diagnosis in the community. ACR Open Rheumatol. (2019) 1:43–51. doi: 10.1002/acr2.1006 31777779 PMC6857982

[4] Martins DF Viseux FJF Salm DC Ribeiro ACA da Silva HKL Seim LA. The role of the vagus nerve in fibromyalgia syndrome. Neurosci Biobehav Rev. (2021) 131:1136–49. doi: 10.1016/j.neubiorev.2021.10.021 34710514

[5] Sarzi-Puttini P Giorgi V Marotto D Atzeni F. Fibromyalgia: an update on clinical characteristics, aetiopathogenesis and treatment. Nat Rev Rheumatol. (2020) 16:645–60. doi: 10.1038/s41584-020-00506-w 33024295

[6] D'Onghia M Ciaffi J Ruscitti P Cipriani P Giacomelli R Ablin JN. The economic burden of fibromyalgia: A systematic literature review. Semin Arthritis Rheumatol. (2022) 56:152060. doi: 10.1016/j.semarthrit.2022.152060 35849890

[7] Ansari AH Pal A Ramamurthy A Kabat M Jain S Kumar S. Fibromyalgia pain and depression: an update on the role of repetitive transcranial magnetic stimulation. ACS Chem Neurosci. (2021) 12:256–70. doi: 10.1021/acschemneuro.0c00785 33397091

[8] Zhang Y Long Y Yu S Li D Yang M Guan Y. Natural volatile oils derived from herbal medicines: A promising therapy way for treating depressive disorder. Pharmacol Res. (2021) 164:105376. doi: 10.1016/j.phrs.2020.105376 33316383

[9] Thompson T Correll CU Gallop K Vancampfort D Stubbs B. Is pain perception altered in people with depression? A systematic review and meta-analysis of experimental pain research. J Pain. (2016) 17:1257–72. doi: 10.1016/j.jpain.2016.08.007 27589910

[10] Leino P Magni G. Depressive and distress symptoms as predictors of low back pain, neck-shoulder pain, and other musculoskeletal morbidity: a 10-year follow-up of metal industry employees. Pain. (1993) 53:89–94. doi: 10.1016/0304-3959(93)90060-3 8316395

[11] Antunes MD da Rocha Loures FCN de Souza IMB Cruz AT de Oliveira Januário P Pinheiro MMLS. A web-based educational therapy intervention associated with physical exercise to promote health in fibromyalgia in Brazil: the Amigos De Fibro (Fibro Friends) study protocol. Trials. (2023) 24:655. doi: 10.1186/s13063-023-07588-3 37814321 PMC10561409

[12] Williams LS Jones WJ Shen J Robinson RL Kroenke K. Outcomes of newly referred neurology outpatients with depression and pain. Neurology. (2004) 63:674–7. doi: 10.1212/01.WNL.0000134669.05005.95 15326241

[13] Hurwitz EL Morgenstern H Yu F. Cross-sectional and longitudinal associations of low-back pain and related disability with psychological distress among patients enrolled in the UCLA Low-Back Pain Study. J Clin Epidemiol. (2003) 56:463–71. doi: 10.1016/S0895-4356(03)00010-6 12812821

[14] Chou K-L. Reciprocal relationship between pain and depression in older adults: evidence from the English Longitudinal Study of Ageing. J Affect Disord. (2007) 102:115–23. doi: 10.1016/j.jad.2006.12.013 17240455

[15] Geerlings SW Twisk JWR Beekman ATF Deeg DJH van Tilburg W. Longitudinal relationship between pain and depression in older adults: sex, age and physical disability. Soc Psychiatry Psychiatr Epidemiol. (2002) 37:23–30. doi: 10.1007/s127-002-8210-2 11926200

[16] Chang M-H Hsu J-W Huang K-L Su T-P Bai Y-M Li C-T. Bidirectional association between depression and fibromyalgia syndrome: A nationwide longitudinal study. J Pain. (2015) 16:895–902. doi: 10.1016/j.jpain.2015.06.004 26117813

[17] Clauw DJ. Fibromyalgia: a clinical review. JAMA. (2014) 311:1547–55. doi: 10.1001/jama.2014.3266 24737367

[18] Giesecke T Williams DA Harris RE Cupps TR Tian X Tian TX. Subgrouping of fibromyalgia patients on the basis of pressure-pain thresholds and psychological factors. Arthritis Rheumatol. (2003) 48:2916–22. doi: 10.1002/art.v48:10 14558098

[19] Al-Hakeim HK Al-Issa AAR Maes M. Serum agrin and talin are increased in major depression while agrin and creatine phosphokinase are associated with chronic fatigue and fibromyalgia symptoms in depression. Metab Brain Dis. (2020) 35:225–35. doi: 10.1007/s11011-019-00506-0 31734845

[20] Smith GD Ebrahim S. 'Mendelian randomization': can genetic epidemiology contribute to understanding environmental determinants of disease? Int J Epidemiol. (2003) 32:1–22. doi: 10.1093/ije/dyg070 12689998

[21] Emdin CA Khera AV Natarajan P Klarin D Zekavat SM Hsiao AJ. Genetic association of waist-to-hip ratio with cardiometabolic traits, type 2 diabetes, and coronary heart disease. JAMA. (2017) 317:626–34. doi: 10.1001/jama.2016.21042 PMC5571980 28196256

[22] Howard DM Adams MJ Shirali M Clarke T-K Marioni RE Davies G. Genome-wide association study of depression phenotypes in UK Biobank identifies variants in excitatory synaptic pathways. Nat Commun. (2018) 9:1470. doi: 10.1038/s41467-018-03819-3 29662059 PMC5902628

[23] Smith DJ Nicholl BI Cullen B Martin D Ul-Haq Z Evans J. Prevalence and characteristics of probable major depression and bipolar disorder within UK biobank: cross-sectional study of 172,751 participants. PLoS One. (2013) 8:e75362. doi: 10.1371/journal.pone.0075362 24282498 PMC3839907

[24] Collins R. What makes UK Biobank special? Lancet. (2012) 379:1173–4. doi: 10.1016/S0140-6736(12)60404-8 22463865

[25] O'Donovan MC. What have we learned from the Psychiatric Genomics Consortium. World Psychiatry. (2015) 14:291–3. doi: 10.1002/wps.20270 PMC4592644 26407777

[26] Kurki MI Karjalainen J Palta P Sipilä TP Kristiansson K Donner KM. FinnGen provides genetic insights from a well-phenotyped isolated population. Nature. (2023) 613:508–18. doi: 10.1038/s41586-022-05473-8 PMC9849126 36653562

[27] Staley JR Blackshaw J Kamat MA Ellis S Surendran P Sun BB. PhenoScanner: a database of human genotype-phenotype associations. Bioinformatics. (2016) 32:3207–9. doi: 10.1093/bioinformatics/btw373 PMC5048068 27318201

[28] Emdin CA Khera AV Kathiresan S. Mendelian randomization. JAMA. (2017) 318:1925–6. doi: 10.1001/jama.2017.17219 29164242

[29] Burgess S Thompson SG Collaboration CCG. Avoiding bias from weak instruments in Mendelian randomization studies. Int J Epidemiol. (2011) 40:755–64. doi: 10.1093/ije/dyr036 21414999

[30] Bowden J Del Greco MF Minelli C Davey Smith G Sheehan NA Thompson JR. Assessing the suitability of summary data for two-sample Mendelian randomization analyses using MR-Egger regression: the role of the I2 statistic. Int J Epidemiol. (2016) 45:1961–74. doi: 10.1093/ije/dyw220 PMC5446088 27616674

[31] Yang J Ferreira T Morris AP Medland SE Madden PAF Heath AC. Conditional and joint multiple-SNP analysis of GWAS summary statistics identifies additional variants influencing complex traits. Nat Genet. (2012) 44:369–75. doi: 10.1038/ng.2213 PMC3593158 22426310

[32] Bowden J Del Greco MF Minelli C Davey Smith G Sheehan N Thompson J. A framework for the investigation of pleiotropy in two-sample summary data Mendelian randomization. Stat Med. (2017) 36:1783–802. doi: 10.1002/sim.v36.11 PMC5434863 28114746

[33] Bowden J Davey Smith G Haycock PC Burgess S. Consistent estimation in Mendelian randomization with some invalid instruments using a weighted median estimator. Genet Epidemiol. (2016) 40:304–14. doi: 10.1002/gepi.2016.40.issue-4 PMC4849733 27061298

[34] Bowden J Davey Smith G Burgess S. Mendelian randomization with invalid instruments: effect estimation and bias detection through Egger regression. Int J Epidemiol. (2015) 44:512–25. doi: 10.1093/ije/dyv080 PMC4469799 26050253

[35] Zhao Q Wang J Hemani G Bowden J Small DS. Statistical inference in two-sample summary-data Mendelian randomization using robust adjusted profile score. Ann Statistics. (2020) 48:1742–69, 28. doi: 10.1214/19-AOS1866

[36] Verbanck M Chen C-Y Neale B Do R. Detection of widespread horizontal pleiotropy in causal relationships inferred from Mendelian randomization between complex traits and diseases. Nat Genet. (2018) 50:693–8. doi: 10.1038/s41588-018-0099-7 PMC6083837 29686387

[37] Burgess S Thompson SG. Interpreting findings from Mendelian randomization using the MR-Egger method. Eur J Epidemiol. (2017) 32:377–89. doi: 10.1007/s10654-017-0255-x PMC5506233 28527048

[38] Loades ME Read R Smith L Higson-Sweeney NT Laffan A Stallard P. How common are depression and anxiety in adolescents with chronic fatigue syndrome (CFS) and how should we screen for these mental health co-morbidities? A clinical cohort study. Eur Child Adolesc Psychiatry. (2021) 30:1733–43. doi: 10.1007/s00787-020-01646-w PMC8558286 32964335

[39] Hubig LT Smith T Williams E Powell L Johnston K Harris L. Measuring interictal burden among people affected by migraine: a descriptive survey study. J Headache Pain. (2022) 23:97. doi: 10.1186/s10194-022-01467-z 35941572 PMC9358846

[40] Valera-Calero JA Úbeda-D'Ocasar E Arias-Buría JL Fernández-de-Las-Peñas C Gallego-Sendarrubias GM Cigarán-Méndez M. Convergent validity of the central sensitization inventory in women with fibromyalgia: association with clinical, psychological and psychophysical outcomes. Eur J Pain. (2022) 26:2141–51. doi: 10.1002/ejp.v26.10 35979630

[41] Shin A Xu H Imperiale TF. The prevalence, humanistic burden, and healthcare impact of irritable bowel syndrome (IBS) among United States veterans. Clin Gastroenterol Hepatol. (2022) 21:1061–9. doi: 10.1016/j.cgh.2022.08.005 PMC9918609 35964894

[42] Thieme K Turk DC Flor H. Comorbid depression and anxiety in fibromyalgia syndrome: relationship to somatic and psychosocial variables. Psychosom Med. (2004) 66:837–44. doi: 10.1097/01.psy.0000146329.63158.40 15564347

[43] Buskila D Cohen H. Comorbidity of fibromyalgia and psychiatric disorders. Curr Pain Headache Rep. (2007) 11:333–8. doi: 10.1007/s11916-007-0214-4 17894922

[44] Henningsen P Zimmermann T Sattel H. Medically unexplained physical symptoms, anxiety, and depression: a meta-analytic review. Psychosom Med. (2003) 65:528–33. doi: 10.1097/01.PSY.0000075977.90337.E7 12883101

[45] Maletic V Raison CL. Neurobiology of depression, fibromyalgia and neuropathic pain. Front Biosci (Landmark Ed). (2009) 14:5291–338. doi: 10.2741/3598 19482616

[46] Alciati A Atzeni F Salaffi F Sarzi-Puttini P. Onset and temporal sequencing patterns of comorbidity between lifetime major depression, panic disorder and fibromyalgia. Clin Exp Rheumatol. (2022) 40:1194–201. doi: 10.55563/clinexprheumatol/ryp027 35699055

[47] Vierck CJ. A mechanism-based approach to prevention of and therapy for fibromyalgia. Pain Res Treat. (2012) 2012:951354. doi: 10.1155/2012/951354 22110947 PMC3200141

[48] Wolfe F Smythe HA Yunus MB Bennett RM Bombardier C Goldenberg DL. The American college of rheumatology 1990 criteria for the classification of fibromyalgia. Report of the multicenter criteria committee. Arthritis Rheumatol. (1990) 33:160–72. doi: 10.1002/art.1780330203 2306288

[49] Wolfe F Clauw DJ Fitzcharles M-A Goldenberg DL Katz RS Mease P. The American College of Rheumatology preliminary diagnostic criteria for fibromyalgia and measurement of symptom severity. Arthritis Care Res (Hoboken). (2010) 62:600–10. doi: 10.1002/acr.20140 20461783

[50] Wolfe F Clauw DJ Fitzcharles M-A Goldenberg DL Häuser W Katz RL. 2016 Revisions to the 2010/2011 fibromyalgia diagnostic criteria. Semin Arthritis Rheum. (2016) 46:319–29. doi: 10.1016/j.semarthrit.2016.08.012 27916278

[51] Arnold LM Bennett RM Crofford LJ Dean LE Clauw DJ Goldenberg DL. AAPT diagnostic criteria for fibromyalgia. J Pain. (2019) 20:611–28. doi: 10.1016/j.jpain.2018.10.008 30453109

[52] Wolfe F Clauw DJ Fitzcharles M-A Goldenberg DL Häuser W Katz RS. Fibromyalgia criteria and severity scales for clinical and epidemiological studies: a modification of the ACR Preliminary Diagnostic Criteria for Fibromyalgia. J Rheumatol. (2011) 38:1113–22. doi: 10.3899/jrheum.100594 21285161

[53] Zachrisson O Regland B Jahreskog M Kron M Gottfries CG. A rating scale for fibromyalgia and chronic fatigue syndrome (the FibroFatigue scale). J Psychosom Res. (2002) 52:501–9. doi: 10.1016/S0022-3999(01)00315-4 12069875

[54] Lawlor DA. Commentary: Two-sample Mendelian randomization: opportunities and challenges. Int J Epidemiol. (2016) 45:908–15. doi: 10.1093/ije/dyw127 PMC5005949 27427429

[55] Liao Q He J Tian F-F Bi F-F Huang K. A causal relationship between leukocyte telomere length and multiple sclerosis: A Mendelian randomization study. Front Immunol. (2022) 13:922922. doi: 10.3389/fimmu.2022.922922 35911771 PMC9337212

[56] Wang T Xu L. Circulating vitamin E levels and risk of coronary artery disease and myocardial infarction: A Mendelian randomization study. Nutrients. (2019) 11:2153. doi: 10.3390/nu11092153 31505768 PMC6770080

