# Supplementary figures and images for: Depression and the risk of fibromyalgia syndrome: a two-sample Mendelian randomization study
Source: Front Psychiatry. 2024 Nov 12;15:1282172. doi: 10.3389/fpsyt.2024.1282172 (PMC11588703; doi:10.3389/fpsyt.2024.1282172)

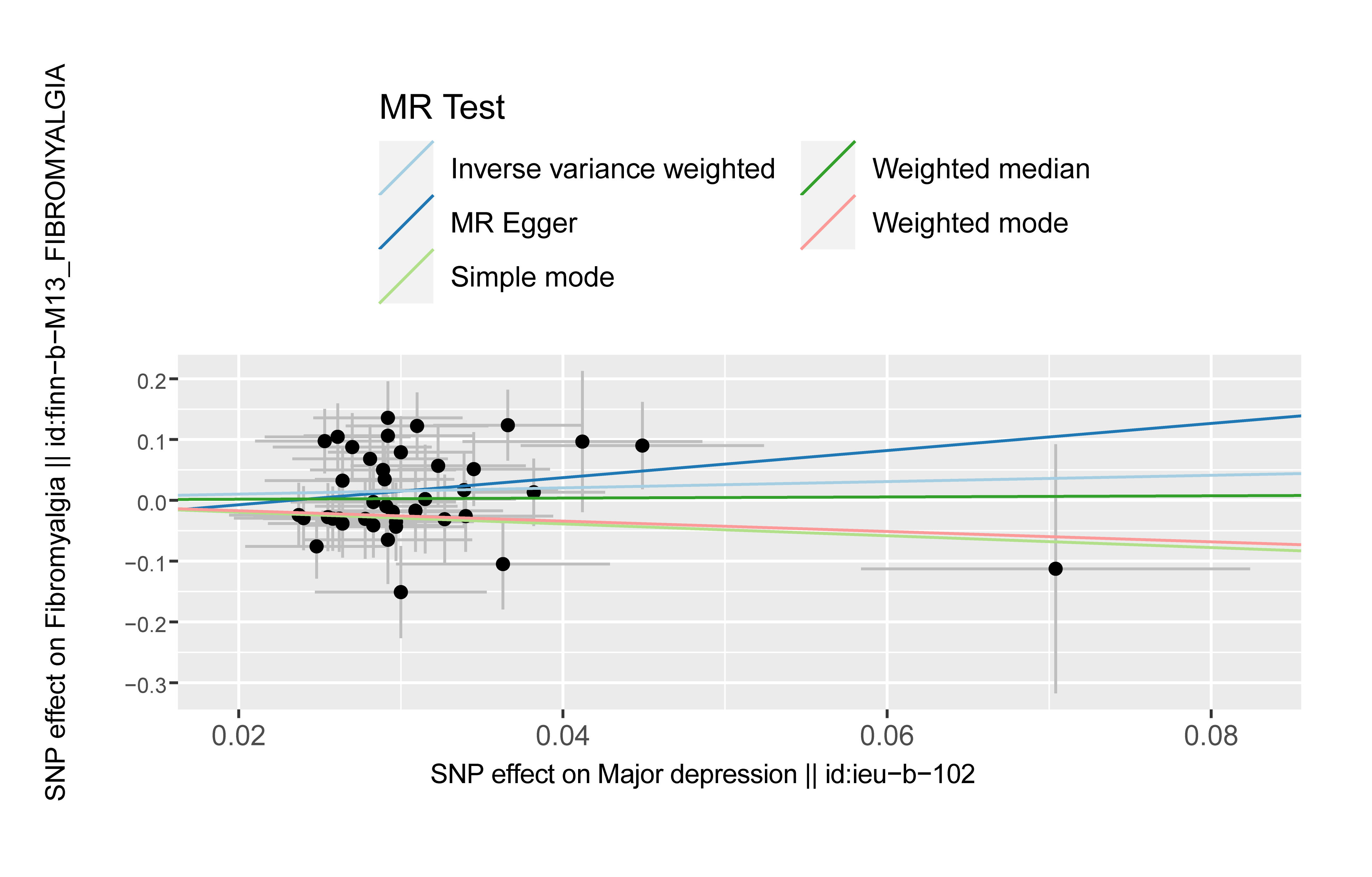

Supplement: Supplementary file 2 [file Image1.tif]

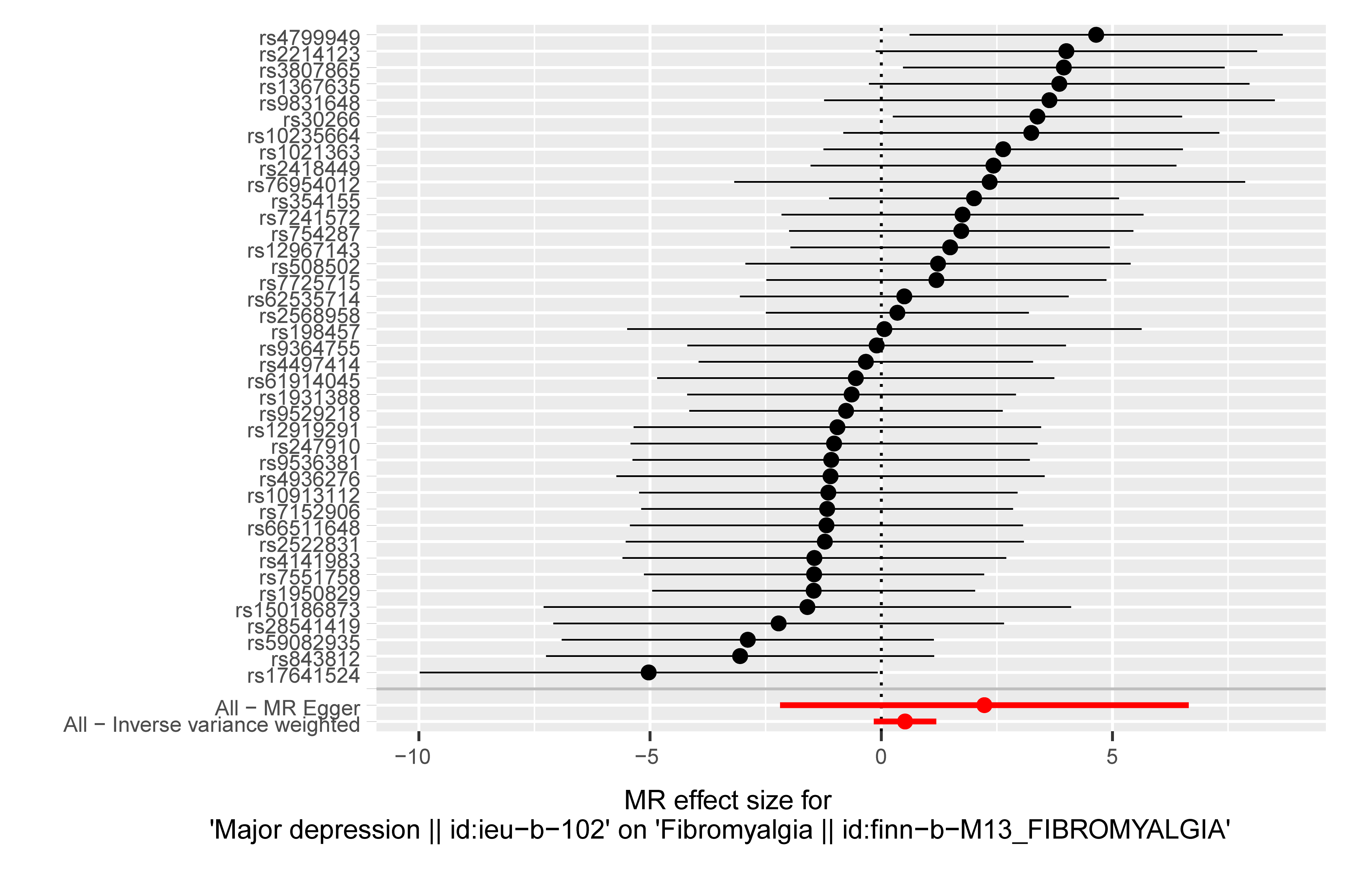

Supplement: Supplementary file 3 [file Image2.tif]

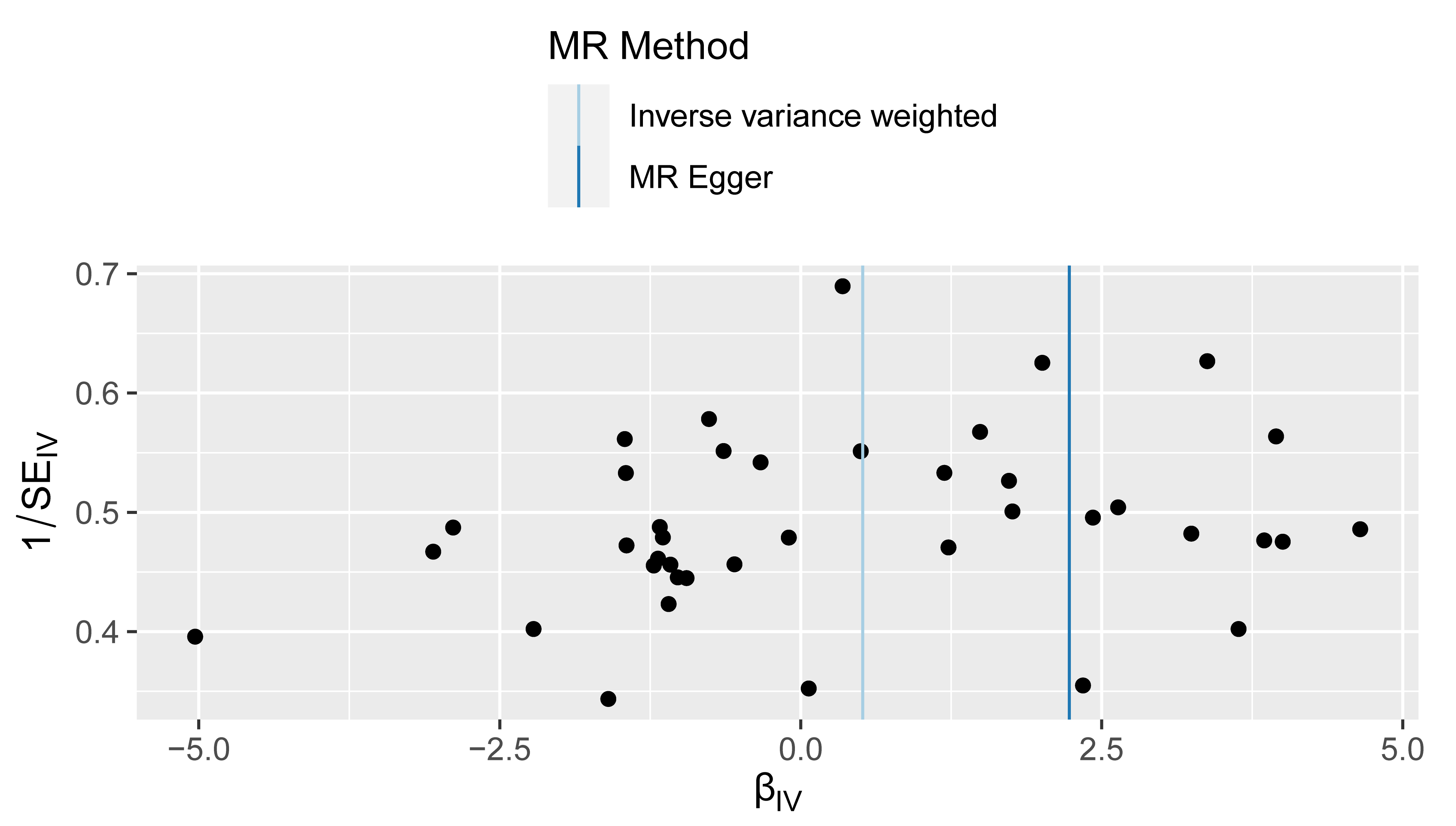

Supplement: Supplementary file 4 [file Image3.tif]

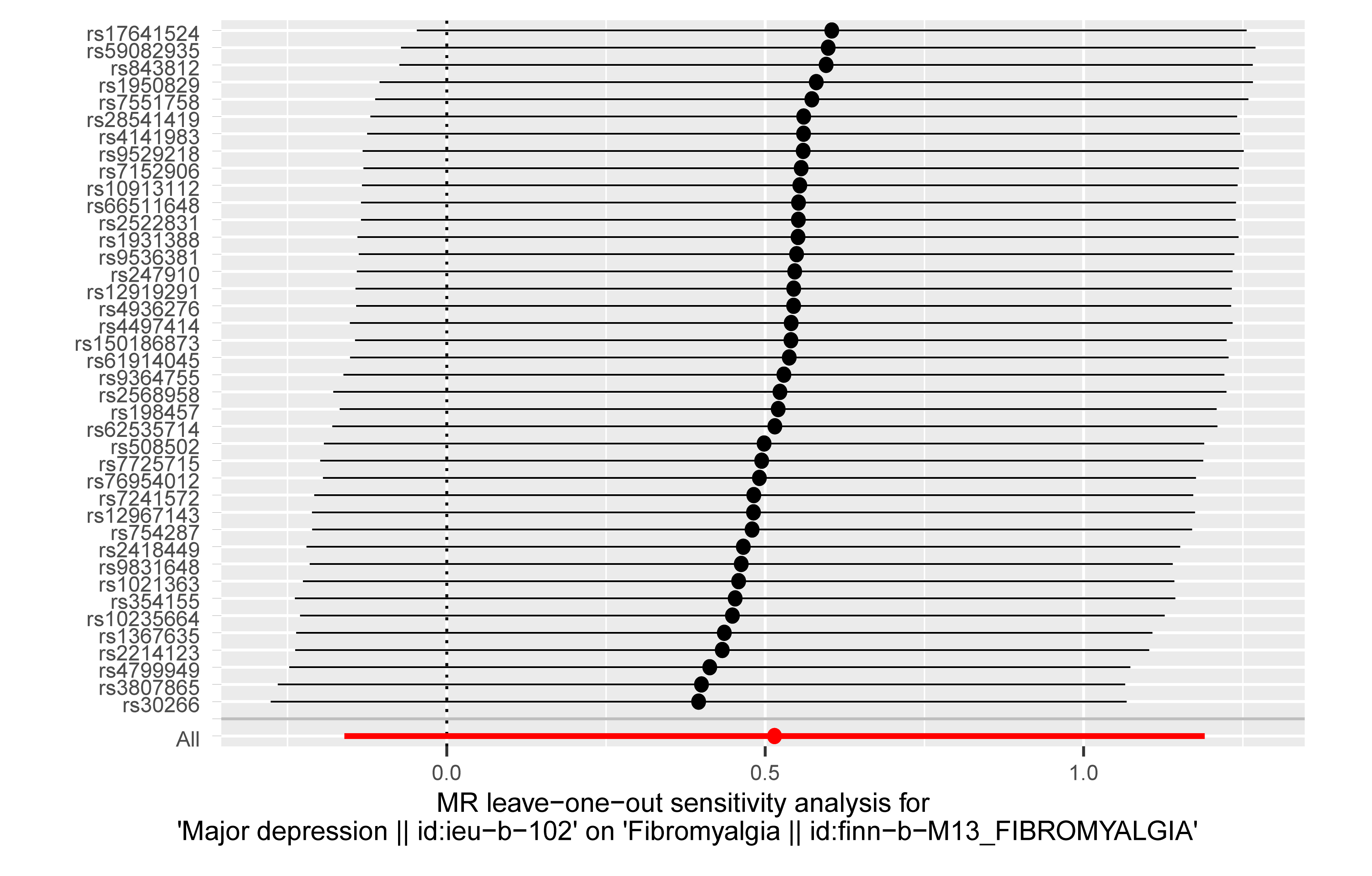

Supplement: Supplementary file 5 [file Image4.tif]
